# A C-Terminal Fragment of Chlorotoxin Retains Bioactivity and Inhibits Cell Migration

**DOI:** 10.3389/fphar.2019.00250

**Published:** 2019-03-20

**Authors:** Mohadeseh Dastpeyman, Paul Giacomin, David Wilson, Matthew J. Nolan, Paramjit S. Bansal, Norelle L. Daly

**Affiliations:** Centre for Molecular Therapeutics, Australian Institute of Tropical Health and Medicine, James Cook University, Cairns, QLD, Australia

**Keywords:** chlorotoxin, tumor-imaging agent, metastasis, migration, invasion, disulfide-rich peptide

## Abstract

Chlorotoxin was originally isolated from the venom of the Israeli scorpion *Leiurus quinquestriatus*, and has potential as a tumor imaging agent based on its selective binding to tumor cells. Several targets have been suggested for chlorotoxin including voltage-gated chloride channels, and it has been shown to have anti-angiogenic activity and inhibit cell migration. The structure of chlorotoxin is stabilized by four disulfide bonds and contains β-sheet and helical structure. Interestingly, the reduced form has previously been shown to inhibit cell migration to the same extent as the wild type, but structural analysis indicates that the reduced form of the peptide does not maintain the native secondary structure and appears unstructured in solution. This lack of structure suggests that a short stretch of amino acids might be responsible for the bioactivity. To explore this hypothesis, we have synthesized fragments of chlorotoxin without disulfide bonds. As expected for such small peptides, NMR analysis indicated that the peptides were unstructured in solution. However, the peptide corresponding to the eight C-terminal residues inhibited cell migration, in contrast to the other fragments. Our results suggest that the C-terminal region plays a critical role in the bioactivity of chlorotoxin.

## Introduction

**Figure 1:**
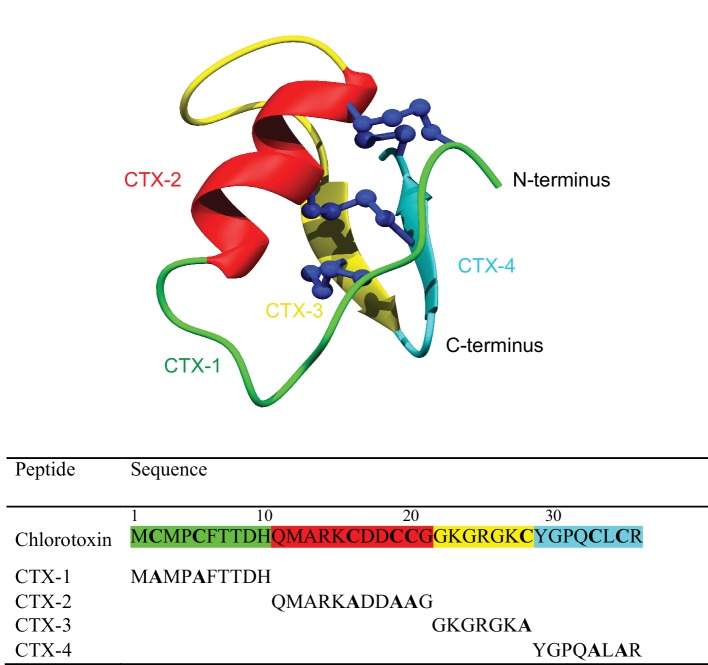
Schematic three-dimensional structure of chlorotoxin (PDB code 5L1C). Each structural element represents the four fragments and is color coded. The sequences for the fragments are given below the structure.

One of the most well-studied scorpion venom peptides is chlorotoxin (CTX), which was originally purified from the venom of the Israeli scorpion *Leiurus quinquestriatus* (DeBin et al., 1993). CTX contains 36 amino acids with 4 disulfide bonds at the core of its well-defined structure. The tertiary structure, as shown in Figure 1, comprises a β-hairpin and an α-helix (Lippens et al., 1995; Correnti et al., 2018). The disulfide connectivity is C_I_–C_IV_, C_II_–C_VI_, C_III_–C_VII_, and C_V_–C_VIII_ (Figure 1).

CTX selectively binds to glioma cells and other embryologically related cells and derived tumors of neuroectodermal origin (Soroceanu et al., 1998; Lyons et al., 2002). By contrast, more than 15 normal human tissues have been shown to be negative for CTX binding (Lyons et al., 2002). The selective binding of CTX to tumor cells bodes well for its application in cancer therapy as a tumor optical imaging contrast agent, particularly for brain cancer (Albert et al., 1994; Kieran et al., 2010; Wiranowska et al., 2011; Kovar et al., 2013; Ojeda et al., 2014). Radiolabeled CTX successfully passed human clinical trial phases I and II (Mamelak et al., 2006; Mamelak and Jacoby, 2007; Gribbin et al., 2009) and FDA approval has been granted to progress to phase III, under the name TM-601 (Lefranc et al., 2018).

CTX is known to inhibit migration and invasion of tumor cells through mechanisms that potentially involve multiple pathways (Soroceanu et al., 1998; Lyons et al., 2002; Ojeda et al., 2014; Dardevet et al., 2015). The molecular targets identified for CTX are all involved in malignant cell migration and invasion, including voltage-gated chloride channels (ClC-3), annexin-2, and matrix metalloproteinase-2 (MMP-2) (Ullrich and Sontheimer, 1996; Deshane et al., 2003; Olsen et al., 2003; Kesavan et al., 2010). It has been shown CTX can effectively block the ClC-3 voltage-gated chloride channel (DeBin and Strichartz, 1991; DeBin et al., 1993), which is selectively expressed in glioma cells (Ullrich et al., 1998; Olsen et al., 2003; Jentsch, 2008). ClC-3 is involved in cell cytoskeleton rearrangements and consequently cell shape and movements during cell migration (Soroceanu et al., 1998; Soroceanu et al., 1999; Mcferrin and Sontheimer, 2006). Furthermore, it has been shown that CTX also interacts with a cell surface protein complex composed of MMP-2, membrane type-I MMP (MT1-MMP), a transmembrane inhibitor of metalloproteinase-2, α_v_β_3_ integrin, and other proteins (Hofmann et al., 2000; Deshane et al., 2003; Jacoby et al., 2010).

Direct binding of CTX with molecular targets has not been experimentally characterized but a recent computational study predicted the binding of CTX with MMP-2 (Othman et al., 2017). The model proposed in this study suggests that the β-sheet of CTX interacts in a region between the collagen binding domain and catalytic domain of the MMP-2, whereas the α-helix of CTX does not appear to be involved in the interaction (Othman et al., 2017).

The three-dimensional structure of disulfide-rich toxins, such as the scorpion venom peptides leiurotoxin-I and charybdotoxin (Drakopoulou et al., 1998; Zhu et al., 2002), is generally critical for bioactivity. However, a recent study suggests this is not the case for CTX. A reduced form of CTX, with the cysteine residues mutated to aminobutyric acid, maintains inhibitory effects on HUVEC cell migration compared to the wild-type peptide, but does not have regular secondary structure (Ojeda et al., 2014). This lack of structure suggests that a short stretch of amino acids might be responsible for the bioactivity. Determining the minimal sequence of CTX with bioactivity is likely to enhance the understanding of the structure/function relationships. We have synthesized four fragments of CTX, containing 7–11 residues, with the cysteine residues replaced with alanine residues to prevent disulfide bond formation, and analyzed the structure, *in vitro* binding, and bioactivity.

## Materials and Methods

### Mammalian Cell Culture

The U-87 MG (ATCC® HTB-14), human primary glioblastoma astrocytoma cell line was grown and maintained in modified Minimum Essential Medium (MEM) (Life Technologies) at 37°C and 5% CO_2_. The growth medium was supplemented to a final concentration of 10% fetal bovine serum (FBS) (Gibco, Scotland), 15 mM HEPES, 1× penicillin-streptomycin solution, 1 mM sodium pyruvate, and 2 mM l-glutamine.

The 1BR.3.GN (ECACC 90020509), human skin normal fibroblast cell line was purchased from the European Collection of Authenticated Cell Cultures (ECACC). 1BR.3.GN cells were grown and maintained in DMEM/F12 (Life Technologies, Australia) containing 1× GlutaMAX, 1× penicillin-streptomycin solution, and supplemented with FBS at 37°C and 5% CO_2_.

### Peptide Synthesis, Purification, and Characterization

Chlorotoxin (CTX) was purchased from Iris Biotech GmbH (Marktredwitz, Germany). CTX fragments were chemically synthesized using standard stepwise Fmoc solid-phase peptide synthesis methods on the rink amide resin (Anaspec, Fremont, CA, USA) using an automated PS3 bench top peptide synthesizer (Protein Technologies, Tuscon, AZ, USA). Peptides were deprotected and cleaved from the resin using the following cleavage cocktail: 95% TFA: 2.5% TIPS: 2.5% H_2_O (v/v/v). The peptide was then precipitated and washed several times with cold diethyl ether, dissolved in 50% acetonitrile:50% H_2_O, lyophilized, and stored as lyophilized powder. Crude peptide mixtures were purified by reversed-phase HPLC (RP-HPLC) on a C_18_ preparative column (Phenomenex Jupiter 250 × 21.2 mm, 10 μm, 300 Å). The peptides were eluted with a 1% gradient from 1 to 60% of solvent Β in solvent A over 60 min at the flow rate of 5 ml/min (Solvent A: 0.05% TFA/water, Solvent B: 0.05% TFA in 90% acetonitrile/water). The purified fractions were characterized by a SCIEX 5800 MALDI TOF/TOF mass spectrometer (SCIEX, Foster city, CA, USA).

### NMR Spectroscopy and Structure Analysis

Samples were prepared from lyophilized peptides at a concentration of 0.2 mM in 90% H_2_O:10% D_2_O with 4,4-dimethyl-4-silapentane-1-sulfonic acid (DSS) as a reference. All NMR spectra were recorded on a 600-MHz AVANCE III NMR spectrometer (Bruker, Karlsruhe, Germany). 2D^1^H-^1^H TOCSY, ^1^H-^1^H NOESY, ^1^H-^15^N HSQC, and ^1^H-^13^C HSQC spectra were collected at 290 K and used for amino acid assignment. All spectra were recorded with an interscan delay of 1 s. NOESY spectra were acquired with mixing times of 200 ms, and TOCSY spectra were acquired with isotropic mixing periods of 80 ms. All spectra were assigned using CCPNMR (Vranken et al., 2005) based on the approach described by Wüthrich (2003).

### Serum Stability Assay

The serum stability of the peptides was tested using human male serum (Sigma, USA), as previously described (Chan et al., 2011). All peptides were tested at a final concentration of 6.6 μM after dilution from a stock solution at 200 μM. The serum was centrifuged at 17,000*g* for 10 min to remove the lipid component, and the supernatant incubated at 37°C for 15 min, prior to conducting the assay. The peptides were incubated in serum or phosphate-buffered saline (PBS) at 37°C, and aliquots were taken at 0, 3, 8, and 24 h (40-μl aliquots were taken in triplicate). The aliquots of serum were quenched with 40 μl of 6 M urea and incubated for 10 min at 4°C. The aliquots were then quenched with 40 μl of 20% trichloroacetic acid (TCA) and incubated for another 10 min at 4°C to precipitate serum proteins. Finally, samples were centrifuged at 17,000*g* for 10 min, and 90 μl of the supernatant was analyzed on reversed-phase HPLC using a Phenomenex Jupiter Proteo C_12_ analytical column (150 mm × 2.00 mm, 4 μm, 90 Å, Phenomenex, Torrance, CA, USA) and a linear gradient of solvent B [90% acetonitrile/10% H_2_O, 0.045% trifluoroacetic acid (TFA)] at a flow rate of 0.4 ml/min using an Agilent 1260 Infinity system (Agilent Technologies, Hanover, Germany). The control samples contained equivalent amounts of the peptides in PBS and were subjected to the same treatment procedure. The stability at each time point was calculated based on the area of the RP-HPLC peaks following incubation with serum at 214 nm as a percentage of the area of 0-h serum-treated peptides (Chan et al., 2013).

### Cytotoxicity Assay

The 2,3-bis-(2-methoxy-4-nitro-5-sulfophenyl)2*H*-tetrazolium-5-carboxanilide (XTT) colorimetry assay was performed to determine whether CTX at the applied concentration was toxic to the U-87 cell line (Page et al., 1993). Cells were seeded in 96-well microplates (7.5 × 10^3^ cells/well) and incubated at 37°C for 24 h. The culture media were then replaced with 150 μl of treatment at two final concentrations of 5 and 60 μM. The treatment was in either media supplemented with 10% FBS or media supplemented with 0.5% FBS, 1× ITS-G, and 1% BSA. DMSO (50% v/v) was used as a control for cell death (Diaz-Perlas et al., 2018). Cells then were incubated at 37°C for 24 h. The media was removed after 24 h and the cells were incubated with 50 μl XTT [1.0 mg/ml in media without phenol red and 0.025 mM phenazine methosulfate (Sigma, USA)] for 3.5 h. Absorbance (A) was measured at 450 nm wavelength, 650 nm used as reference wavelength in a plate reader (Polarstar Optima, Germany). Cell viability was calculated as percentage of control cells using the equation: (A_450_ − A_650_) of treated cells × 100/(A_450_ − A_650_) of control cells.

The experiments were repeated three times in pentaplicates and statistical significance of the observed difference was determined using one-way ANOVA with Dunnett’s multiple comparison tests using the GraphPad Prism v7 software (GraphPad Software Inc., USA).

### Peptide Biotinylation

CTX fragments were biotinylated on the resin to be detected by streptavidin-APC for the binding assay according to the following protocol. The assembled peptide on the resin (0.1 mmol) was washed with DMF. Biotin (Sigma, USA) was coupled to the N-terminal amide using HCTU/DIPEA activation in DMSO:DMF (1:1), overnight. The resin was washed with DMSO:DMF (1:1) twice to remove excess biotin and washed further with DMF (2×) and DCM (2×) and allowed to dry before proceeding to cleavage step. The biotinylated peptide was cleaved from the resin as previously mentioned. The mono-labeled peptides were then purified using preparative RP-HPLC and characterized using a SCIEX 5800 MALDI TOF/TOF mass spectrometer (SCIEX, Foster City, CA, USA) and analytical RP-HPLC.

The CTX biotinylation was performed as described previously (Selo et al., 1996). Briefly, 1 mg/ml CTX (Iris, Germany) solution was prepared in phosphate buffer (pH 8.0) and incubated with 27.5 μl of 10 mM NHS-Biotin (Sigma, USA) in DMSO (10 molar excess). The final concentration of the organic solvent was kept <20% in the reaction buffer. After 6 h of incubation at room temperature, excess biotin was removed by passing the reaction solution through Phenomenex Strata C_8_ unit (55 μm, 70 Å). The collected sample was characterized using TripleTOF® 6,600 Quadrupole Time-Of-Flight (QTOF) mass analyzer (SCIEX, Foster City, CA, USA) and analytical RP-HPLC.

### Cell Surface Binding and Internalization Assay

Flow cytometric analysis was performed to investigate binding of CTX and fragments to U-87 MG and 1BR.3.GN cells. For cell surface binding analysis, cells were detached from adherent culture using StemPro™ Accutase™ Cell Dissociation reagents (Gibco, USA), washed and resuspended in media supplemented with 0.5% FBS, 1× ITS-G, and 0.1% BSA. After adjusting the cell density to 10^6^ cells per ml, 100 μl of the cell suspension was placed in round-bottom 96-well plate. For each concentration of CTX and fragments, duplicate wells received treatments of either 5 or 50 μM CTX or CTX fragments for 30 min at 4°C. Biotinylated anti-mouse/human CD44 antibody (BioLegend) was used as control positive in all sets of experiments (Knupfer et al., 1999).

Following the incubation, cells were washed 3 times with PBS and centrifuged at 400 g for 3 min to remove the unbound peptides. APC-conjugated streptavidin (BD Pharmingen, USA) was added at a concentration of 6.5 μg/ml to each well and incubated for further 30 min on ice. Incubation was followed by two further washes in PBS and stained cells were then analyzed on a BD FACS Canto II flow cytometer (BD Bioscience, USA). Data analysis was performed using FlowJo software (Tree Star, v. 8.8.4.).

To investigate whether CTX fragments internalize into cells *via* the same thermodynamic pathway as CTX, cells were incubated with CTX fragments at a concentration of 50 μM in 0.5% media supplemented with 1× ITS-G and 0.1% BSA for 30 min at 37°C, with 5% CO_2_. Then cells were washed with PBS and fixed using 1× fixation/permeabilization buffer (eBioscience, USA). Cells were washed and permeabilized using 1× permeabilization buffer (eBioscience, USA) for 10 min. Cells then were stained with APC-streptavidin at 4°C for 30 min. Samples were washed twice with PBS and subsequently analyzed on BD FACS Canto II flow cytometer (BD Bioscience, USA) and data were analyzed as described above.

### Cell Migration/Invasion Assay

Migration assays were performed using CytoSelect cell migration assay kit (8 μm, Fluorometric format, Cell Biolabs, USA) according to the manufacturer’s instructions. Briefly, cells were serum-starved overnight, then harvested using StemPro™ Accutase™ Cell Dissociation reagents and resuspended in media supplemented with 1× ITS-G and 1% BSA at 10^6^ cells/ml. Aliquots of 100 μl of cells were then pre-incubated with CTX or fragments (at two concentrations of 5 and 50 μM) for 30 min at room temperature. Then cells were added to each insert (upper chamber), and 150-μl media supplemented with or without 10% FBS (chemoattractants) was added to each well (lower chamber). The plate was incubated at 37°C for 16 h to allow cell migration through the polycarbonate membrane pores in response to chemoattractant (10% FBS) and subsequently lysed and detected by CyQuant® GR dye. Fluorescence measurement was performed using a fluorescence plate reader (Polarstar Optima, Germany) with a 485/520-nm filter (at an extinction of 480 nm and an emission of 520 nm). The percentage inhibition of migration in each treatment was calculated relative to that of the untreated cells. Statistical analyses using one-way ANOVA were performed for comparing treatments against control cells followed by Dunnett’s test using the GraphPad Prism software.

The invasion assay was performed using CytoSelect cell invasion assay kit (8 μm, Fluorometric format, Cell Biolabs, USA) using the same protocol. The cells were allowed to pass through a polycarbonate membrane bearing 8-μm-sized pores with a thin layer of basement membrane for 24 h. Cells that had invaded to the other side of the inserts were lysed and detected by CyQuant® GR dye. The percentage inhibition of invasion in each treatment was calculated relative to that of the untreated cells. Statistical analyses using one-way ANOVA were performed for comparing treatments against control cells followed by Dunnett’s test using the GraphPad Prism software.

### Protein Array Analysis of CTX

CTX used for the protein array analysis was chemically synthesized using Fmoc chemistry with 2-chlorotrityl resin and oxidized using 0.1 M ammonium bicarbonate pH 8 and 1 mM reduced glutathione, with stirring overnight at room temperature. The peptide was biotinylated using a 20-fold excess of EZ-Link® Sulfo-NHS-LC-biotin (ThermoFisher Scientific), according to the manufacturer’s instructions. The biotinylated sample was analyzed utilizing an SCIEX 5800 MALDI TOF/TOF mass spectrometer (SCIEX, Foster city, CA, USA), followed by purification of labeled CTX (to remove excess non-reacted and hydrolyzed biotin reagent from the solution) *via* HPLC. The purified sample was dried and resuspended in 100 μl of 1.0% PBS (pH 7.2) to give a final concentration of 10 μg. Protein-protein interactions among biotinylated CTX and more than 9,000 human proteins were then investigated employing a ProtoArray® Human Protein Microarray v5.0 Protein-Protein Interaction (PPI) kit (Invitrogen™) and significant interactions detected employing a GenePix® microarray scanner (Molecular Devices), according to the manufacturer’s instructions.

## Results

### Design and Synthesis of Chlorotoxin Fragments

To determine the minimal sequence requirements of CTX to elicit cellular effects, four CTX fragments (CTX-1, -2, -3, -4) were chemically synthesized using Fmoc solid-phase peptide synthesis. The sequences of the full-length CTX and synthetic fragments, and the location of the fragments in the CTX structure are shown in Figure 1. The crude peptides were purified using RP-HPLC and mass analysis carried out using MALDI TOF/TOF mass spectrometry.

### Structural Analysis With NMR Spectroscopy

The structures of the purified peptides were analyzed using NMR spectroscopy. The one-dimensional spectra of the CTX fragments did not have significant dispersion in the amide region. Further analysis of the TOCSY and NOESY spectra for the individual fragments allowed assignment of the resonances and analysis of the secondary shifts, which indicated that the peptides had shifts close to random coil, consistent with the peptides being unstructured in solution (Supplementary Figure S1).

### Serum Stability Assay

**Figure 2:**
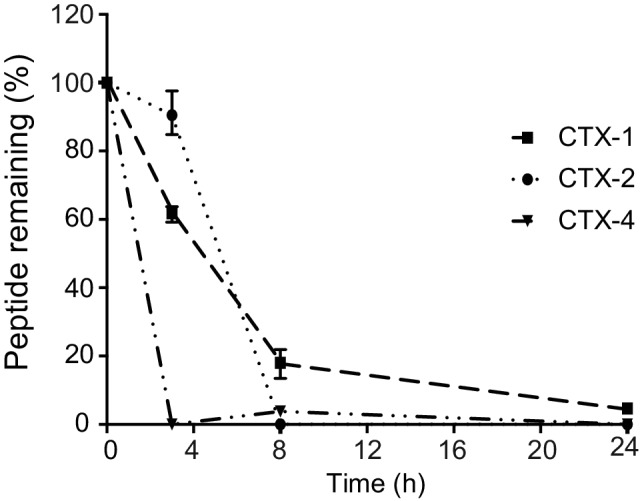
Stability of the peptides in human serum. The amount of peptide remaining was calculated by comparing the area of the elution peak of peptides incubated in human serum and that of peptides that had not been incubated. The experiments were performed in triplicate. All data are represented as mean ± SEM.

The stability of the CTX peptide fragments in human serum was assessed using RP-HPLC, and the degradation profiles are shown in Figure 2. CTX-1 and -2 degraded more slowly than CTX-4. The latter peptide had less than 10% peptide remaining after 3 h, whereas CTX-1 had more than 18% remaining at the 8-h time point and could be detected up to 24 h. The serum stability could not be measured accurately for CTX-3 peptide, using the same column as that used for the other fragments, because of the hydrophilic nature of this peptide. CTX has been previously shown to be highly stable in serum with only 10% degradation after 24-h incubation with human serum at 37°C using the same protocol (Akcan et al., 2011; Diaz-Perlas et al., 2018).

Due to low serum stability of CTX fragments, the cell assays were designed to minimize the exposure of the peptides to serum media (FBS). Prior to the cell assays, cells were pre-treated with CTX or CTX fragments in 0.5–1% media supplemented with 1× ITS-G and 0.1% BSA which were used to ensure cells remain healthy and viable over the course of the 30-min treatment (Gstraunthaler, 2003). It has been previously shown that CTX achieves the maximum cellular level at a concentration of 10 μM within 30 min of incubation with cells under physiological conditions (37°C, 5% CO_2_) (Wiranowska et al., 2011). CTX has been shown to significantly label cells (Kesavan et al., 2010), block chloride channel (Mcferrin and Sontheimer, 2006), inhibit endothelial cell migration (Ojeda et al., 2014), and induce biochemical changes in U87-MG cells (Falahat et al., 2016) over 30-min incubation with cells. Thus, 30 min of pre-treatment appears to be the optimum treatment time for CTX and fragments, in this study.

### Cytotoxicity Assay

The cytotoxicity of CTX and the CTX fragments was investigated using an XTT assay on the U-87 MG cell line. Cells were incubated with either 5 or 60 μM peptide. These concentrations are comparable to those used in the subsequent cell assays. The XTT assays were carried out in media (10%), and also in media supplemented with 0.5% FBS, 1× ITS-G, and 1% BSA. The CTX fragments were not cytotoxic to U87-MG cells under either media condition or peptide concentration, with cell viability after a 24-h incubation period higher than 90% for all peptides tested (Supplementary Figure S2).

These results are consistent with previous study that showed CTX is not toxic to U87-MG cells and HUVECs at concentrations up to 10 and 200 μM, respectively (Ojeda et al., 2014; Park et al., 2018).

### Peptide Biotinylation

Peptides were labeled with biotin to allow detection using flow cytometry. The CTX fragments were biotinylated on resin, resulting in a single biotin moiety attached to the N-terminus. Following incubation with biotin, the peptides were purified using RP-HPLC to remove excess biotin. By contrast, CTX was biotinylated in solution. The mass spectrometry analysis indicated the presence of CTX species with one, two, three, and four biotin moieties attached, consistent with the four biotinylation sites. Excess biotin was removed from the sample using RP-HPLC but it was not possible to purify the individual species, and therefore, the mixture was used in the subsequent experiments.

### Cell Surface Binding/Endocytosis Assay

**Figure 3:**
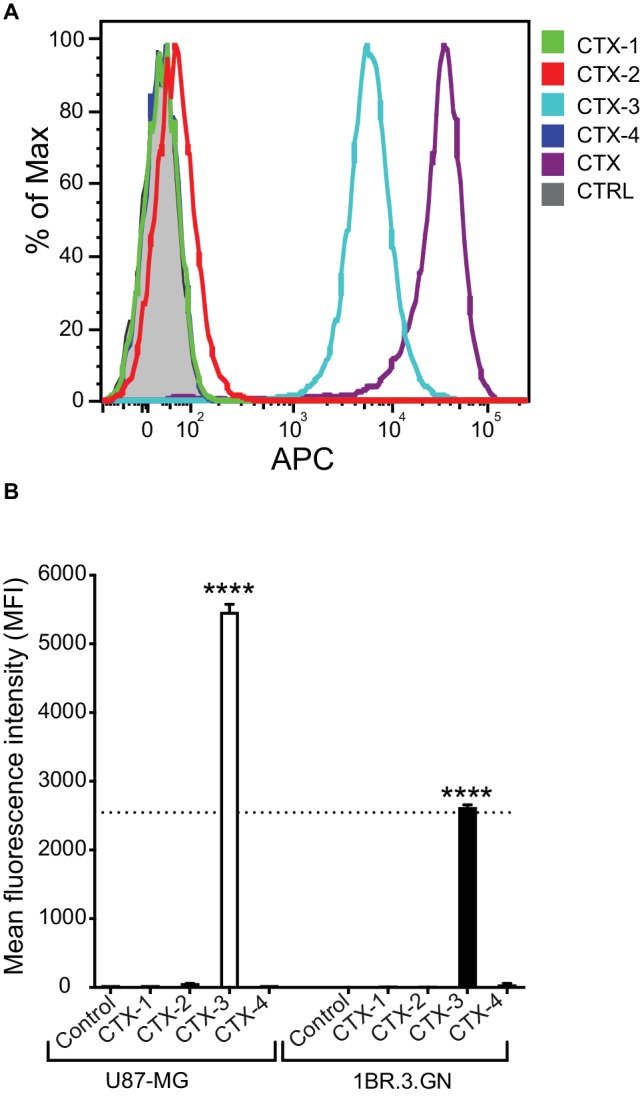
*In vitro* cell surface binding of CTX fragments. **(A)** Representative flow cytometric histogram of *in vitro* U87-MG cell surface binding of biotinylated CTX fragments detected by APC-labeled streptavidin. Cells were treated with 50 μM CTX or fragments at 4°C for 30 min. Gray shaded histograms represent cells treated with APC-streptavidin alone. **(B)** Mean fluorescence intensity (MFI) of *in vitro* cell surface binding of CTX fragments (50 μM) to U87-MG and 1BR.3.GN cell line. The experiments were performed three times in duplicate. Data are presented as mean ± SEM. The statistical analysis was performed using an ANOVA Dunnett’s multiple comparison test by comparison of CTX fragments with control cells (****p < 0.0001). The data were normalized to account for differences in cell number and expressed as percent of maximum cell number for each sample (% of Max).

To evaluate the interaction of CTX fragments with U87-MG cells, a cell line previously shown to bind to CTX, *in vitro* binding assays were carried out with the biotinylated peptides and binding detected by flow cytometry using APC-conjugated streptavidin as shown in Figure 3A. As expected, native CTX demonstrated a significant level of APC fluorescence and surface binding to cells when compared to control-treated cells (Figure 3A). Further analysis of the relative binding efficiency of the four fragments revealed that while CTX-1, CTX-2, and CTX-4 did not display any significant binding to the surface of cells, CTX-3 did significantly bind to the surface of both U87-MG and 1BR.3.GN cell lines in a dose-dependent manner (Figures 3A,B). These studies were done at 4°C. A direct comparison of the peptides with CTX is difficult, given the differences in the number of biotin moieties present in the full-length peptide and the fragments, which will affect the signal intensity.

**Figure 4:**
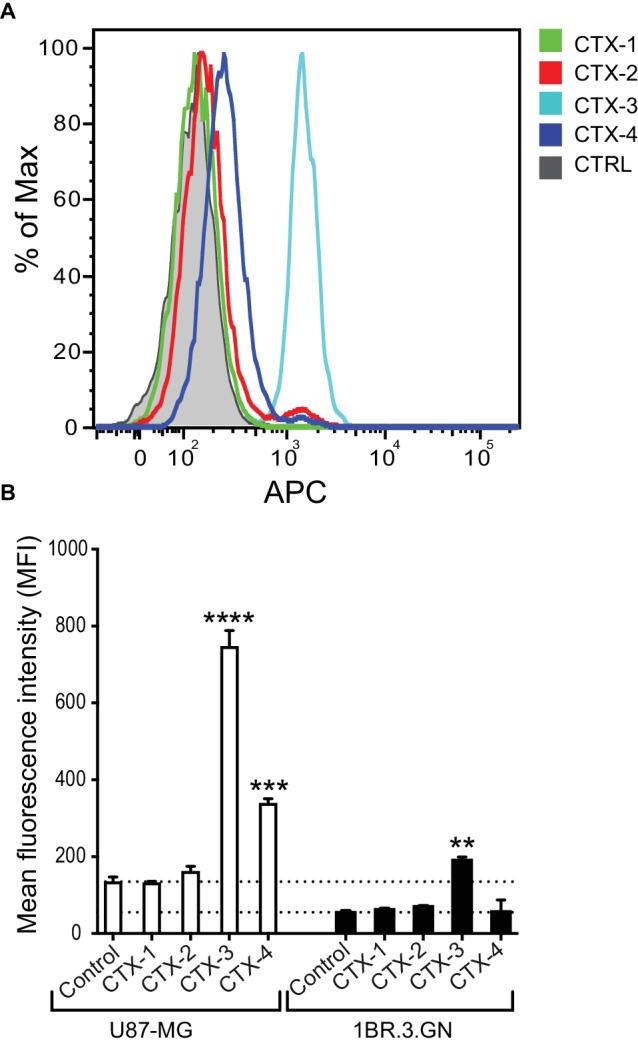
*In vitro* cell internalization of CTX fragments. **(A)** Representative flow cytometric histogram of *in vitro* U87-MG cell internalization of biotinylated CTX fragments detected by APC-labeled streptavidin. Cells were treated with 50 μM CTX fragments at 37°C for 30 min. Gray shaded histograms represent cells treated with APC-streptavidin alone. (**B)** Mean fluorescence intensity (MFI) of *in vitro* cell internalization of CTX fragments (50 μM) to U87-MG and 1BR.3.GN cell line. The experiments were performed three times in duplicate. Data are presented as mean ± SEM. The statistical analysis was performed using an ANOVA Dunnett’s multiple comparison test by comparison of CTX fragments with control cells (**p < 0.01, ***p < 0.001, ****p < 0.0001).

We next assessed whether the peptide fragments were being internalized and interacting with a target within the cell. Analysis of the internalization of the fragments into the U87-MG cells at 37°C indicated that while CTX-1 and CTX-2 do not appear to be internalized into either the tumor cell line (U87-MG) or normal cell line (1BR.3.GN), CTX-3 and CTX-4 appeared to be binding internally to U87-MG cells (Figures 4A,B). Interestingly, only CTX-3 showed any evidence of fluorescence signal when exposed to 1BR.3.GN cells; however, it is not clear whether this binding was on the surface of the cell or internal (Figure 4B). Given that CTX-4 appeared to bind internally to U87-MG cells but not 1BR.3.GN cells suggests that this intracellular interaction may be specific to the glioblastoma cell line. Chlorotoxin has been previously shown not to bind to human fibroblasts (Lyons et al., 2002).

### Migration and Invasion Assay

**Figure 5:**
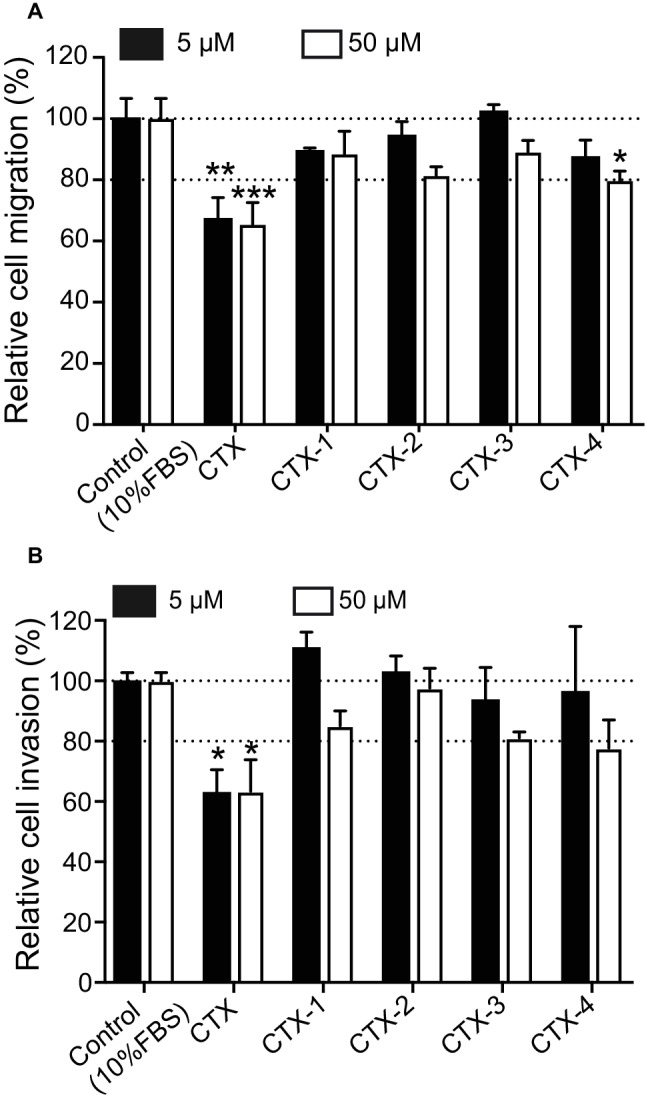
The migration and invasion assay. Cells were pre-incubated with CTX and fragments at 5 and 50 μM and then were used for migration/invasion assay. **(A)** The effect of CTX and fragments on U87-MG cell migration. **(B)** The effect of CTX and fragments on U87-MG cell invasion. The percentage inhibition of migration/invasion in each treatment was calculated relative to that of the untreated well. The experiments were performed two times in quadruplicate. The data were expressed as mean ± SEM. Data were analyzed by one-way ANOVA against peptide control. *p < 0.05, **p < 0.01, ***p < 0.001.

The inhibitory effects of CTX and the CTX fragments on migration and invasion of U87-MG cells were analyzed and the results summarized in Figure 5. CTX significantly inhibited U87-MG cell migration with inhibitory effects of 32.9% (*p* < 0.01) and 34.6% (*p* < 0.001) compared to control cells at 5 and 50 μM, respectively (Figure 5A). Only CTX-4 inhibited U87-MG cell migration at a concentration of 50 μM (20.4%, *p* < 0.05).

CTX also significantly inhibited U87-MG cell invasion by 37% (*p* < 0.05) and 36.7% (*p* < 0.05) at 5 and 50 μM (Figure 5B), respectively, consistent with previous studies (Deshane et al., 2003; Mcferrin and Sontheimer, 2006; Kesavan et al., 2010). None of the CTX fragments significantly inhibited U87-MG cell invasion even at the highest concentration tested (50 μM).

### Protein Array Analysis of CTX

Protein-protein interactions among biotinylated CTX and more than 9,000 human proteins were investigated employing a ProtoArray® Human Protein Microarray v5.0 Protein-Protein Interaction (PPI) kit (Invitrogen™) and monitored with a GenePix® microarray scanner (Molecular Devices). Of the more than 9,000 possible interactions tested herein, 104 (ca. 1.0%) were significant (*Z*-score of >3), while 4 of the top 9, including interactions between CTX and cortactin (CTTN; Z-score 32.27), polo-like kinase 1 (PLK1; Z-score 11.20), homeobox protein HOXB6 (Z-score 10.47), and XTP3-transactivated protein A (XTP3TPA; *Z*-score 6.21825), are relevant to the present investigation as these proteins have been shown to be involved in cancer.

## Discussion

Given the potential of CTX as a therapeutic, it is of significant interest to understand the structural features important for function, as this information is likely to facilitate the development of CTX as a therapeutic. Numerous studies have analyzed the binding of CTX and conjugates to cells but there is still limited information on the structure-function relationships and target interaction.

The CTX fragments synthesized in the current study showed limited stability in human serum and did not display regular secondary structure in solution. Interestingly, CTX-2 was the most stable of the peptides tested in human serum and is also the longest peptide, which might explain the enhanced stability. The lack of structure in solution is expected for such small peptides and is consistent with the previous study, which showed that the disulfide bonds in CTX are critical for the three-dimensional structure (Ojeda et al., 2014).

The C-terminal fragments of CTX (CTX-3 and CTX-4) displayed interesting properties in the cellular assays. CTX-3 showed cell surface binding and cell internalization, whereas CTX-4 was internalized into cells and was the only fragment to inhibit U87-MG cell migration at the highest concentration tested (50 μM). Despite the effect of CTX-4 on cell migration, it did not inhibit cell invasion.

The cell surface binding and internalization of CTX is critical to its potential as a tumor imaging agent and our current results suggest that residues 22–28, present in CTX-3, might be involved with this process. However, given that CTX-3 binds to both a tumor cell line and a normal cell line, in contrast to previous studies on CTX, it appears likely that this region is not sufficient by itself to account for the selective binding to tumor cells. It is possible the cell surface binding observed for CTX-3 is non-specific membrane binding mediated by the presence of the high proportion of positively charged (two lysine and one arginine) residues within a seven-residue peptide as reported for other highly charged peptides (Futaki et al., 2007; Ojeda et al., 2017). Although no cell surface binding was observed for CTX-4, it was the only fragment that appeared to be specifically internalized into the glioma cells at 37°C. The differences between CTX-3 and CTX-4 suggest they might have different internalization mechanisms.

Our results, whereby CTX-4 only inhibits migration and not invasion, indicate that it might inhibit the ClC-3 channel, which is involved in cell migration, independently from the MMP2 macromolecular complex, which is involved in cell invasion. The other possibility is that CTX-4 inhibits U87-MG cell migration through another pathway, which does not necessarily involve the macromolecular complex. Annexin A2, which is identified as another CTX target, is involved in cell migration in a series of glioma cells including U87-MG cells (Tatenhorst et al., 2006; Kesavan et al., 2010). Therefore, it is possible that CTX-4 inhibits U87-MG cell migration through an annexin A2 interaction. However, more research, including mutagenesis studies, are required to investigate CTX-4 binding to the targets previously suggested for CTX. Additional studies involving the synthesis of over-lapping peptide fragments might also provide further insight into the structure/function relationships.

The recently proposed model of MMP-2 interacting with CTX (Othman et al., 2017) shows that residues 29–36 (which correspond to CTX-4) are directly involved in the interaction with MMP-2 (Othman et al., 2017). Neither of the cysteine residues (C33 and C35) in this region interact with MMP-2, therefore it is highly likely that replacing cysteines with alanine in our study would not make a significant difference in CTX-4 binding and bioactivity. Furthermore, a recent study has used CTX as inspiration to design blood-brain barrier shuttles. MiniCTX3 contains residues 28–32 of CTX and a lactam bridge, and was able to transport nanoparticles across endothelial cells (Diaz-Perlas et al., 2018). These studies highlight the potential importance of the C-terminal region of CTX in cellular interactions and support our current findings.

Although several biological targets for CTX have been proposed, direct binding has not been reported. Here, we used protein array technology to identify another possible target for CTX. CTX bound most strongly to cortactin, a substrate of the Src nonreceptor tyrosine kinase. Overexpression of cortactin is linked to invasive cancers, including melanoma, colorectal cancer, and glioblastoma, making it an important biomarker for invasive cancers (MacGrath and Koleske, 2012). Cortactin localizes to invasive protrusions in cells including invadopodia and has been shown to associate with MMP-2 using co-immunoprecipitation studies (Banon-Rodriguez et al., 2011). Similar to annexin A2, it has been suggested that a transient interaction between cortactin and complexes containing MMPs might exist (Banon-Rodriguez et al., 2011). Given CTX can penetrate cells *via* clathrin-mediated endocytosis, it is possible that it can interact with cortactin intracellularly, or alternatively as part of a MMP-2 complex that is subsequently internalized (Wiranowska et al., 2011). The importance of intracellular targeting of cortactin has been shown with a cell-permeable peptide that binds cortactin, and inhibits the invasion of a range of cancers including glioblastomas (Hashimoto et al., 2006). Our cell internalization assay provides support for the possibility that CTX-4 can bind an intracellular target that is specific to tumor cells compared to normal cells. It would be of interest to determine if CTX-4 can also bind to cortactin.

Overall, this study provides insight into understanding the important sequence requirements for CTX bioactivity, and indicates that a small region of CTX, which is not structured in solution, can have an influence on cell migration, albeit at relatively high concentrations. This result might explain the bioactivity previously observed for the reduced form of CTX (Ojeda et al., 2014). We also provide evidence that fragments of CTX are involved in cell binding and/or internalization. Further study is required to determine if these fragments can bind to the biological targets previously suggested for CTX.

## Data Availability

The datasets generated for this study are available on request to the corresponding author.

## Author Contributions

ND and MD contributed to study conception. MD, MN, and PB performed experiments. MD, ND, PG, and MN contributed to data analysis. MD, ND, and PG contributed to data presentation. MD and ND contributed to manuscript preparation. MD, ND, DW, and PG contributed to manuscript editing. ND, DW, and PG contributed to supervision. ND contributed to funding acquisition.

### Conflict of Interest Statement

The authors declare that the research was conducted in the absence of any commercial or financial relationships that could be construed as a potential conflict of interest.

## Non-Standard Abbreviations

ANOVA: Analysis of variance
APC: Allophycocyanin
BSA: Bovine serum albumin
CTX: Chlorotoxin
DIPEA: Ethyldiisopropylamine
DMSO: dimethyl sulfoxide
DSS: 4,4-dimethyl-4-silapentane-1-sulfonic acid
ECACC: European Collection of Authenticated Cell Cultures
FBS: Fetal bovine serum
HCTU: 2-(6-chloro-1-H-benzotriazole-1-yl)-1,1,3,3-tetramethylaminium hexafluorophosphate
HEPES: 4-(2-hydroxyethyl)-1-piperazineethanesulfonic acid
HSQC: Heteronuclear single quantum correlation experiment
HUVEC: Human umbilical vein endothelial cells
ITS-G: Insulin-transferrin-selenium
MALDI TOF/TOF MS: Matrix-assisted laser desorption/ionization-time of flight mass spectrometry
MEM: Minimum essential medium
MMP-2: MMP matrix metalloproteinase-2
NHS: N-hydroxy-succinimide
NMR: Nuclear magnetic resonance
NOESY: Nuclear overhauser spectroscopy
PBS: Phosphate-buffered saline
QTOF: Time-of-flight mass spectrometry
RP-HPLC: Reversed-phase HPLC
TCA: Trichloroacetic acid
TFA: Trifluoroacetic acid
TOCSY: Total correlated spectroscopy
XTT: 2,3-Bis(2-methoxy-4-nitro-5-sulfophenyl)-2*H*-tetrazolium-5-carboxanilide inner salt.
