# Supplementary material for: A C-Terminal Fragment of Chlorotoxin Retains Bioactivity and Inhibits Cell Migration
Source: Front Pharmacol. 2019 Mar 20;10:250. doi: 10.3389/fphar.2019.00250 (PMC6435586; doi:10.3389/fphar.2019.00250)
Supplement: Supplementary file 1 [file Table_1.DOCX]

**Supplementary information:**

**Figure S1**


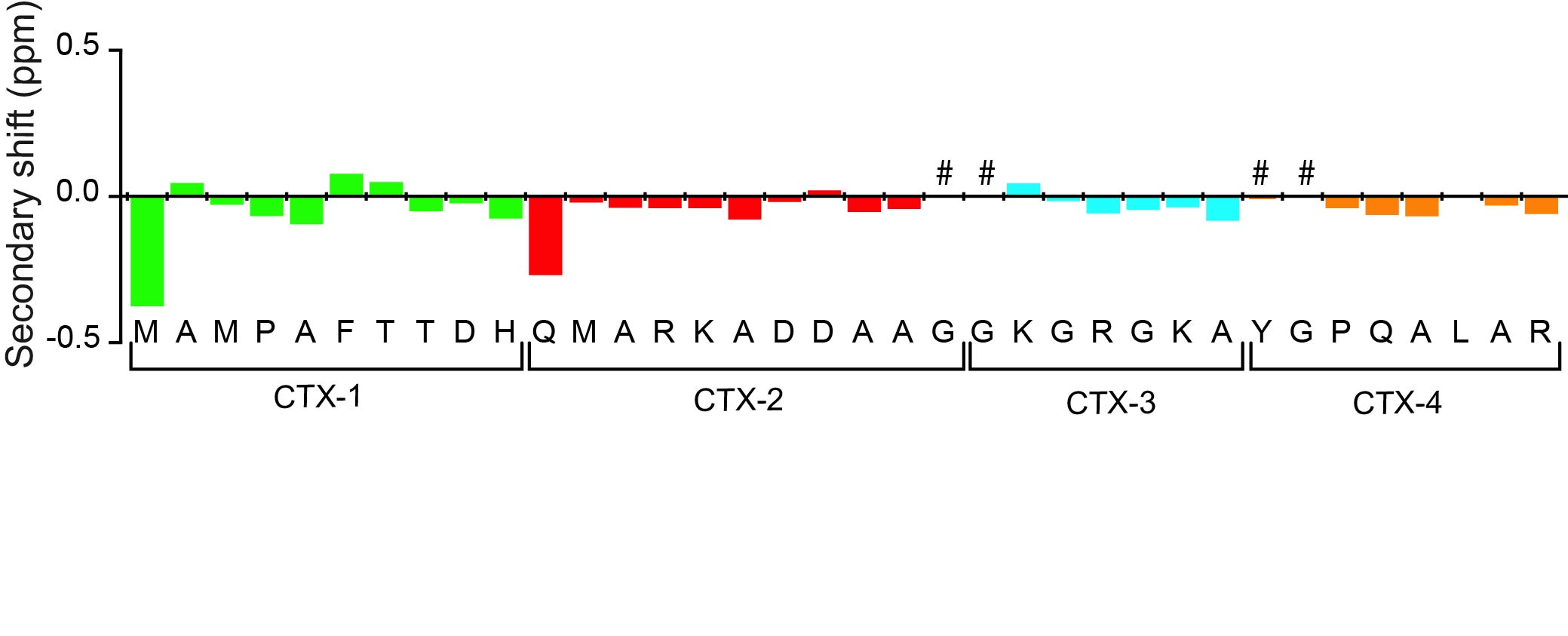


**Secondary shifts of CTX fragments.** The secondary shifts were obtained by subtracting random coil shifts from the αH shifts ([Wishart et al., 1995](#_ENREF_46)). **(#:** could not be assigned)

**Figure S2:**


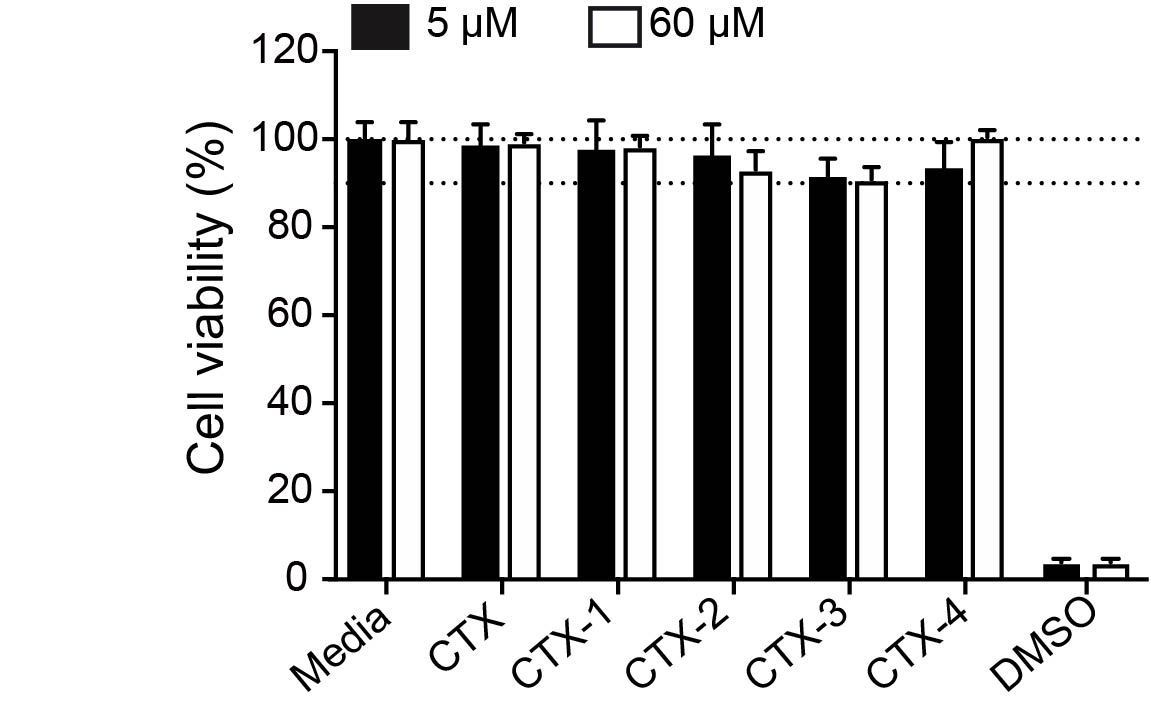


**The cell viability of CTX and fragments to U87-MG cells after 24 h.** CTX and fragments were added in media supplemented with 0.5% FBS, 1x ITS-G and 1% BSA. Cell viability was determined by XTT assay. Controls with media and DMSO at 50% (v/v) were used to establish 100 and 0% of cell survival, respectively. The experiments were repeated three times with 5 replicates. Data are shown as mean ± SEM.
